# The association of the anesthesiologist’s academic and educational status with self-confidence, self-rated knowledge and objective knowledge in rational antibiotic application

**DOI:** 10.1186/s13104-020-05010-8

**Published:** 2020-03-18

**Authors:** Frederick Schneider, Christian M. Schulz, Matthias May, Gerhard Schneider, Christian Ernst, Matthias Jacob, Kai Zacharowski, Thomas Hachenberg, Maren Schmidt, Moritz Kretzschmar, Bernhard Graf, Martin G. Kees, Michael Pawlik, Michael Sander, Christian Koch, Michael Zoller, Markus Heim

**Affiliations:** 1 Department of Anaesthesiology and Intensive Care, Technical University of Munich, TUM School of Medicine, Klinikum rechts der Isar, Ismaninger Str. 22, 81675 Munich, Germany; 2 Department of Urology, Klinikum St. Elisabeth Straubing GmbH, St.-Elisabeth Straße 23, 94315 Straubing, Germany; 3 Department of Anesthsiology, Klinikum St. Elisabeth Straubing GmbH, St.-Elisabeth-Straße 23, 94315 Straubing, Germany; 4 Department of Anesthesiology, Intensive Care Medicine and Pain Therapy, University Hospital Frankfurt, Theodor-Stern-Kai 7, 60590 Frankfurt Am Main, Germany; 5 Department of Anesthesiology and Intensive Care Medicine, Otto-von-Guericke-University Magdeburg, Leipziger Str. 44, 39120 Magdeburg, Germany; 6 Department of Anesthesiology and Intensive Care Medicine, Klinikum Barnim GmbH, Werner Forßmann Krankenhaus, Rudolf-Breitscheid-Straße 100, 16225 Eberswalde, Germany; 7 Department of Anesthesiology, University Hospital Regensburg, Franz-Josef-Strauß-Allee 11, 93053 Regensburg, Germany; 8 Department of Anesthesiology, Caritas St. Josef Medical Center, University of Regensburg, Landshuter Straße 65, 93053 Regensburg, Germany; 9 Department of Anesthesiology, Operative Intensive Care Medicine and Pain Therapy, University Hospital of Giessen and Marburg, Rudolf-Buchheim-Straße 7, 35392 Gießen, Germany; 10 Department of Anesthesiology, Hospital of the University of Munich, LMU Munich, Marchioninistr. 15, 81377 Munich, Germany

**Keywords:** Antibiotics, Multi-resistant pathogens, Microbiological diagnostics, Anti-infective therapy, MR2, Education, Anesthesia

## Abstract

**Objective:**

This study aimed to investigate the association of anesthetists’ academic and educational status with self-confidence, self-rated knowledge and objective knowledge about rational antibiotic application. Therefore, anesthetists in Germany were asked about their self-confidence, self-rated knowledge and objective knowledge on antibiotic therapy via the Multiinstitutional Reconnaissance of practice with Multiresistant bacteria (MR2) survey. Other analysis from the survey have been published elsewhere, before.

**Results:**

361 (52.8%) questionnaires were completed by specialists and built the study group. In overall analysis the Certification in Intensive Care (CIC) was significantly associated with self-confidence (p < 0.001), self-rated knowledge (p < 0.001) and objective knowledge (p = 0.029) about antibiotic prescription. Senior consultant status was linked to self-confidence (p < 0.001) and self-rated knowledge (p = 0.005) but not objective knowledge. Likewise, working on Intensive Care Unit (ICU) during the last 12 months was significantly associated with self-rated knowledge and self-confidence (all p < 0.001). In a logistic regression model, senior consultant status was not associated with any tested influence factor. This analysis unveiled that CIC and working on ICU were more associated with anesthesiologists’ self-confidence and self-rated knowledge than senior consultant status. However, neither of the characteristics was thoroughly associated with objective knowledge.

## Introduction

Work experience has repeatedly been correlated with improved performance in psychological research [1], as well as anesthetic practice [2, 3]. However, performance varied to a greater extent within a group of the same experience level than between groups of different experience levels [4, 5]. Together with experience, knowledge is recognized to be one of the main influence factors for clinical decision making [6–9].

Beyond knowledge, self-confidence is an important influence factor on decision-making [10]. Junior doctors showed low levels of self-confidence; [11, 12] this is important, since perceived confidence had a significant effect on clinical behavior [12, 13]. Hale and colleagues examined nurses’ knowledge and self-confidence regarding evidence-based antibiotic use [14]. Despite high levels of baseline self-confidence, they identified knowledge deficits and misperceptions that might result in adverse events [14].

In anesthesia antibiotics are routinely given either as a part of perioperative microbial prophylaxis or when treating severe infections on the intensive care unit. It is unknown whether senior consultant status, an additional certification in intensive care medicine (CIC) or current practice on the intensive care unit (ICU) enhances performance most. Therefore, the present analysis aimed to investigate the association of CIC, senior consultant status, and time spent on ICU with anesthesiologists’ self-confidence, self-rated knowledge and objective knowledge with respect to the rational use of antibiotics. For this purpose, we analyzed the Multiinstitutional Reconnaissance of practice with MultiResistant bacteria (MR2) survey [15–17]. This analysis extends published research evaluating gender differences [16], disparities between university and non-university physicians [17], and between specialists and non-specialists [15] in self-confidence about rational antibiotic application.

## Main text

### Materials and methods

#### Study setting

In 2017, the MR2 questionnaire containing 55 items about rational antibiotic application was distributed to sixteen anesthesiologic departments in Germany. Seven university, one primary care, six secondary care and two tertiary care hospitals participated in the study. The Bavarian medical association’s Ethics Committee waived the need for ethics approval and written informed consent (Registration number: 18-040; Bayerische Landesärztekammer, BLÄK). The MR2 trial was designed as a survey study. Anesthetists were requested to fill a questionnaire about their self-confidence and self-rated knowledge regarding their daily work with antibiotics. Given substances, antibiotic regimens and doses were not evaluated as a part of the study. Also, no antibiotic or antimicrobial medication was requested as a part of or given because of the study protocol. In order to reach a significance of p < 0.05 and a margin of error of less than 5%, a sample size of 379 respondents was determined based on the number of registered anesthesiologists in Germany in 2016 (23.531) [18].

#### Development of the survey

The first version of the MR2-Survey has been developed 2015 in order to evaluate self-confidence and knowledge about rational antibiotic application among physicians from different medical specialties in Germany [19]. Questions were verbalized following a thorough literature review and the consultation of specialists for infectious diseases [19]. In 2017, this version of the MR2-Survey was adapted to specifically include anesthetic problems. [19–23] Besides five demographic questions, the query contained items on the participant’s self-confidence (n = 6, Likert scale; 1 = very unconfident, 2 = unconfident, 3 = confident, 4 = very confident), self-rated knowledge (n = 16, Likert scale; 1 = no knowledge, 2 = little knowledge, 3 = knowledge, 4 = full knowledge), and objective knowledge (n = 5, multiple choice) regarding multi-resistant pathogens and the rational use of antibiotics. In line with prior research about self-confidence in a medical environment, four-point Likert-Scales were chosen so that participants had to decide between the positive and negative axes [24, 25]. Twenty–three further items evaluated the participant’s opinion about problems in context with antibiotic use (n = 13, results not shown), as well as hospital standards and personal preferences on perioperative antibiotic application (n = 10, results not shown).

#### Data analysis

The retrieved questionnaires were centrally scanned and tested for plausibility. In order to reach a data integrity of more than 94%, only queries that contained more than 52 answered items were included to the analysis. Participating departments were requested to provide data about organizational details like supervision of the intensive care unit, availability of departmental guidelines on perioperative antibiotic prophylaxis, and number of employees with a diploma in antibiotic stewardship (ABS, obtainable through a course of 160 h). Furthermore, they were asked to specify local *E. coli* ciprofloxacin resistance rates and the local rate of *MRSA* (multi-resistant staphylococcus aureus) for the year 2016.

#### Academic and educational status

Only specialists in anesthesiology (curriculum of 5 years including 1 year of intensive care medicine) were included into the study group for the present analysis. Besides the sole time an individual spent on the ICU during the last 12 months, two academical and educational characteristics were analyzed: First, the presence of an additional certification in intensive care (CIC), which can be obtained after spending at least one additional year in intensive care medicine. Second, the academic status of being either senior consultant or head of department. In anesthesiologic departments in Germany, consultants are–in contrast to specialists in anesthesiology—likely to be supervisor of several operation theatres or a large number of ICU-patients.

#### Statistical analysis

For the single-item comparison between participants with or without the respective attribute, Kruskal–Wallis tests were conducted for ordinal variables, while Chi Square tests were used to compare nominal variables. The Kruskal–Wallis test was chosen, since we could not postulate a normal distribution within all analyses. A detailed description of applied tests and compared categories can be found in the footnotes of the respective table. To investigate the associations of CIC, senior consultant status, and time spent on intensive care unit during the last 12 months with the items of the survey, a multivariate logistic regression model (LRM) was designed. The LRM accounted for (1) CIC, (2) senior consultant status, (3) time the individual spent on the intensive care unit during the last 12 months, (4) number of self-contained indications for antibiotic application during the last 7 workdays before the study, and (5) participants gender. In order to generate binary variables, for self-confidence, the answers very-unconfident and unconfident (1 and 2) were tested against confident and very confident (3 and 4), for self-rated knowledge, no-knowledge and little-knowledge (1 and 2) were compared against knowledge and full-knowledge (3 and 4) [17, 26]. For the analysis of objective knowledge correct answers were tested against incorrect answers or no answer [16, 17]. For self-confidence and self-rated knowledge all calculations were also made after pooling the items. For objective knowledge the overall number of correct answers was compared between the groups. Here, individuals with more than 60% correct answers were compared to those with less; in line with the 60% success criterion throughout the medical curriculum in Germany [27, 28]. SPSS Statistics 24.0 (IBM Corp., Armonk, NY, USA) was used for statistical analysis and significance was defined as p < 0.05.

#### Results

684 questionnaires were returned from sixteen anesthesiologic departments in Germany (medium 45 employees, inter-quartile range 32–105) and met the inclusion criteria (53.9%). Of these, 323 were completed by residents, so that 361 queries (52.8%) filled by specialists in anesthesiology were included into the analysis. Table 1 presents descriptive characteristics for the study group. Further details about the study population can be found elsewhere [15].

**Table 1:** Descriptive characteristics of the study group

	Study group (n=361)	100% time spent on ICU (n=52)	50–99% time spent on ICU (n=60)	1–50% time spent on ICU (n=113)	No time spent on ICU (n=135)
Educational status					
Specialist in anesthesiology	205 (56.8%)	22 (42.3%)	26 (43.3%)	66 (58.4%)	91 (66.9%)
Specialist with CIC^a^	156 (43.2%)	30 (57.7%)	34 (56.7%)	47 (41.6%)	45 (33.1%)
Academic position					
Specialist	206 (57.2%)	22 (42.3%)	38 (63.3%)	67 (59.3%)	79 (58.5%)
Senior consultant or head of department	154 (42.8%)	30 (57.7%)	22 (36.7%)	46 (40.7%)	56 (41.5%)
Number of patients with self-contained anti-infective treatment during the last 7 days					
No patient (Option 1)	92 (25.8%)	3 (5.8%)	7 (11.9%)	32 (28.6%)	50 (37.3%)
1–2 patients (Option 2)	64 (17.9%)	12 (23.1%)	16 (27.1%)	24 (21.4%)	12 (9.0%)
3–5 patients (Option 3)	57 (16.0%)	6 (11.5%)	12 (20.3%)	18 (16.1%)	21 (15.7)
> 5 patients (Option 4)	144 (40.3%)	31 (59.6%)	24 (40.7%)2	38 (33.9%)	51 (38.1%)
Number of employees with additional certification in ABS (in the department)^b^					
No employee (Option 1)	234 (64.8%)	36 (69.2%)	38 (63.3%)	75 (66.4%)	85 (62.5%)
One employee (Option 2)	96 (26.6%)	14 (26.9%)	18 (30.0%)	31 (27.4%)	33 (24.3%)
Two employees (Option 3)	21 (5.8%)	1 (1.9%)	1 (1.7%)	2 (1.8%)	17 (12.5%)
Three Employees (Option 4)	10 (2.8%)	1 (1.9%)	1 (1.7%)	5 (4.4%)	1 (0.7%)
Gender					
Female	119 (33.8%)	19 (38.8%)	16 (27.6%)	27 (24.1%)	57 (42.9%)
Male	295 (81.7%)	39 (75.0%)	51 (85.0%)	103 (91.2)	102 (75.0%)
Administration of intensive care unit^b^					
Only anesthesia	295 (81.7%)	39 (75.0%)	51 (85.0%)	103 (91.2)	102 (75.0%)
Shared administration	66 (18.3%)	13 (25.0)	9 (15.0%)	10 (8.8%)	34 (25.0%)
Knee arthroplasties done at the hospital^b^					
No	52 (14.4)	5 (9.6%)	9 (15.0%)	21 (18.6%)	17 (12.5%)
Yes	309 (85.6%)	47 (90.4%)	51 (85.0%)	92 (81.4)	119 (87.5%)
Colorectal surgeries done at the hospital^b^					
No	0	0	0	0	0
Yes	361 (100%)	52 (100%)	60 (100%)	113 (100%)	136 (100%)
Specific hospital or departmental guidelines for anti-infective prophylaxis available^b^					
No	71 (19.7%)	10 (19.2%)	11 (18.3%)	16 (14.2%)	34 (25.0%)
Yes	290 (80.3%)	42 (80.8%)	49 (81.7%)	97 (85.8%)	102 (75.0%)

Descriptive statistics for the study group as well as after separation for occupation ratio in the Intensive Care Unit (ICU) during the last 12 working months before the survey. Data are provided as number and percentages within the respective group (vertically)

^a^The German physicians can obtain an additional certification post residency after one additional year of full-time work on an intensive care unit

^b^Hospital related data that were assigned to each participant

#### Factors associated with anesthesiologists’ self-confidence about antibiotics

An additional certification in intensive care (CIC) was significantly associated with anesthesiologists’ self-confidence about the rational use of antibiotics (p < 0.001; Table 2) in the overall analysis. While time spent on ICU during the last 12 months itself was associated with self-confidence (p < 0.001), there was no obvious effect of the amount of time an individual spent on ICU in terms of varying odds-ratios (all p < 0.005). These results were consistent in the logistic regression (p = 0.003 for CIC and p < 0.001 for time spent on ICU, Table 2). Senior consultant status, however, was significantly associated with self-confidence in the overall analysis (p < 0.001), but not in the LRM (p = 0.098). Additional file 1: Table S1 presents the results of the single-item analysis.

**Table 2:** Overall associations of academic and educational influence factors on self-confidence, self-rated knowledge, and objective knowledge

	Certificate in intensive care (CIC)					Senior consultant status					Time spent on ICU								
	Non CIC (Mean ± SD)	CIC (Mean ± SD)	p^a^	OR CIC (95% CI)	p-MLRM^b^	Non senior (Mean ± SD)	Senior (Mean ± SD)	p^a^	OR Senior (95% CI)	p-MLRM^b^	Non ICU Mean ± SD)	ICU Mean ± SD)	P^a^	1–50% ICU OR (95% CI)	p-MLRM^b^	50–100% OR (95% CI)	p-MLRM^b^	100% OR (95% CI)	p-MLRM^b^
Self-confidence	2.59 ± 0.45	2.97 ± 0.42	< 0.001**	2.52 (1.37–4.65)	0.003*	2.65 ± 0.41	2.92 ± 0.51	< 0.001**	1.67 (0.91–3.05)	0.098	2.52 ± 0.46	2.91 ± 0.42	< 0.001**	4.73 (2.55–8.77)	< 0.001**	4.45 (1.90–9.99)	< 0.001**	4.55 (1.86–11.09)	0.001*
Self-rated knowledge	2.59 ± 0.45	2.96 ± 0.44	<0.001**	3.62 (1.90-6.90	<0.001**	2.63 ± 0.40	2.91 ± 0.54	0.005*	1.17 (0.64–2.14)	0.617	2.47 ± 0.43	2.90 ± 0.44	<0.001**	2.92 (1.63–5.25)	<0.001**	4.89 (2.07–11.59)	<0.001**	11.55 (3.33–40.00)	<0.001**
Objective knowledge	3.17/5 ± 1.0	3.38/5 ± 1.08	0.029*	1.60 (0.96–2.68)	0.74	3.23/5 ± 0.97	3.20/5 ± 1.12	0.265	0.91 (0.54–1.51)	0.708	3.17/5 ± 1.08	3.32/5 ± 0.10	0.638	1.06 (0.62–1.81)	0.823	0.3 (0.42–1.62)	0.579	1.20 (0.60–2.41)	0.605

Results from the comparison of means and the logistic regression adjusted for the following criteria (1) additional certification in intensive care, (2) senior consultant status, (3) work on the intensive care unit within 12 preceding months, (4) self-contained anti-infective medication during 7 preceding workdays, (5) participants gender; Item-wise comparisons have been computed using the Kruskal–Wallis Test. The Kruskal–Wallis Test as well as the MLRM compare the self-confidence levels very unconfident and unconfident vs confident and very confident for self-confidence, no knowledge and little knowledge vs. knowledge and full-knowledge for self-rated knowledge as well as individuals with more than 60% correct answers vs. others for objective knowledge, respectively. The German physicians can obtain an additional certification post residency after one additional year of full-time work on an intensive care unit The German physicians can obtain an additional certification post residency after one additional year of full-time work on an intensive care unit

All values are provided as mean ± standard deviation (SD) for participants with the respective attribute. For objective knowledge the reference refers to the mean number of questions (/5) answered correctly. (a) p-values for unadjusted comparisons; (b) *p* value from the logistic regression model

*ICU* intensive care unit, *SD* standard deviation, *OR* Odd’s ratio, *CI* confidence interval, *P-LRM* p-values gathered from the LRM

*p < 0.05

**p < 0.001

#### Factors associated with anesthesiologists’ self-rated knowledge about antibiotics

Likewise self-confidence, anesthetists with a CIC rated their knowledge higher (2.96 ± 0.43) than those without (2.59 ± 0.45, p < 0.001). Additionally, senior consultant status (p = 0.005) and time spent on ICU (p < 0.001) lead to higher self-rated knowledge in the comparison of means. Here, in contrast to self-confidence, the more time the anesthetist spent on ICU during the last year, the stronger was the association with self-rated knowledge about the rational use of antibiotics (all p < 0.001; see Table 2 for details). Interestingly, the correlation of senior consultant status with self-rated knowledge was not reproducible in the LRM (p = 0.617). Results of the single-item analysis are presented in Additional file 2: Table S2.

#### Factors associated with anesthesiologists’ objective knowledge about antibiotics

In the overall analysis of objective knowledge about antibiotic application, people with a CIC (mean 3.38 correct answers) differed significantly from those without (mean 3.17 correct answers, p = 0.029). This was not consistent in the LRM (p = 0.74). Beyond, neither senior consultant status, nor time spent on ICU were associated with objective knowledge in the LRM or simple comparisons (all p > 0.005; see Table 2 for details). Additional file 3: Table S3 provides further information about differences between the groups with respect to the single item analysis.

### Discussion

The aim of this study was to investigate the association of anesthesiologists’ self-confidence, self-rated knowledge and objective knowledge about rational antibiotic with their educational and academic background. Both, the overall and the single item analysis unveiled an association of the CIC with self-confidence and self-rated knowledge (Additional file 1: Table S1 and Additional file 2: Table S2). Except in the comparison of anesthesiologists with or without CIC, objective knowledge, was not correlated with any of the tested independent variables.

In the analysis of self-rated knowledge, the association with 100% time spent on the ICU was prominent in contrast to the remaining influence factors (p < 0.001). Thus, we assume that time spent working in intensive care is related to self-confidence and self-rated knowledge about antibiotics, while senior consultant status is not. In detail, specialists with a CIC esteemed themselves significantly more self-confident and rated their knowledge significantly higher (see Table 2 for details).

Elsewhere, fellowship background and training influenced objective knowledge scores in different medical settings [29, 30]. Regarding antibiotics, in contrast, educational actions significantly increased self-confidence but not knowledge [13]. However, we assume that a background of one additional training year in intensive care (CIC) should increase knowledge about these topics. Accordingly, the mean number of correct answers significantly differed between anesthetists with a CIC and these without (p = 0.029, Table 2 for details). Nevertheless, objective knowledge was not associated with spent on ICU nor CIC were. This might be influenced by the design of our knowledge questions: They rather tested knowledge on perioperative practice, than profound knowledge about the rational use of antibiotics. Notwithstanding, the TARRAGONA strategy recommends to “listen to your hospital” in order to prompt a rational antibiotic therapy [31]. Thus, in our opinion, specialists (physicians with a CIC, or anesthetists working on the ICU) are supposed to have a more sophisticated knowledge about local resistance patterns.

In the comparison of means, senior consultant status significantly increased self-confidence (p < 0.001) and self-rated knowledge (p = 0.005). Its association with more organizational aspects (e.g. local resistance patterns) in the single item analysis (Additional file 2: Table S2) might reflect the additional organizational and supervisory duties a consultant needs to satisfy. In line, Al Hadi and colleagues were not able to detect knowledge differences between consultants and specialists in their research on knowledge about transcranial magnetic stimulation [32]. Likewise, Bashiri and co-workers found no influence of pediatric subspecialisation on knowledge about the management of febrile seizures [33]. Nevertheless, both groups detected significant knowledge differences between consultants and other physician groups (e.g. residents) [32, 33].

## Limitations

- We did not investigate surrogates of the participant’s experience (e.g. working years) and hence, we cannot rule out that differences in self-confidence, self-rated knowledge and objective knowledge between the groups are a consequence of greater experience within one or another group.
- The general problems with survey studies also apply for the MR2-query: a response rate of 53.9% might have biased the results, since very uncertain individuals as well as seniors with a high-certainty might have been missed (non-response bias) and respondents might have responded as expected from them (social-desirability-response-set).
- The evaluation of self-confidence and self-rated knowledge used a four-point Likert-Scale that did not include a dimension for uncertainty or neutrality. This might bias the results since one side of the scale might appear better or worse to the participants in absence of a defined neutral reference category.
- The MR2 survey only included five items on objective knowledge and therefore, it might not have sufficient validity for the detection of discrete knowledge differences.

## Supplementary information

**Additional file 1: Table S1.** Associations of the MR2′s items on self-confidence with certification in Intensive care, senior consultant status, and ratio of occupation on ICU. This table presents the results of the respective single-item statistical analysis of the MR2′s items on self-confidence and their association with certification in Intensive care, senior consultant status, and ratio of occupation on ICU.

**Additional file 2: Table S2.** Associations of the MR2’s items on self-rated knowledge with certification in Intensive care, senior consultant status, and ratio of occupation on ICU. This table presents the results of the respective single-item statistical analysis of the MR2’s items on self-rated knowledge and their association with certification in Intensive care, senior consultant status, and ratio of occupation on ICU.

**Additional file 3: Table S3.** Associations of the MR2’s items on objective knowledge with certification in Intensive care, senior consultant status, and ratio of occupation on ICU. This table presents the results of the respective single-item statistical analysis of the MR2’s items on objective knowledge and their association with certification in Intensive care, senior consultant status, and ratio of occupation on ICU.

## Data Availability

The datasets used and analyzed during the current study are available from the corresponding author on reasonable request.

## Abbreviations

LRM: Logistic regression model
ABS: Antibiotic stewardship
CIC: Certification as intensive care physician
ICU: Intensive care unit
MR2: Multiinstitutional reconnaissance of practice with multiresistant bacteria
MRSA: Multiresistant staohylococcus aureus
