# Supplementary material for: The association of the anesthesiologist’s academic and educational status with self-confidence, self-rated knowledge and objective knowledge in rational antibiotic application
Source: BMC Res Notes. 2020 Mar 18;13:161. doi: 10.1186/s13104-020-05010-8 (PMC7079461; doi:10.1186/s13104-020-05010-8)
Supplement: Supplementary file 1 — Additional file 1: Table S1. Associations of the MR2′s items on self-confidence with certification in Intensive care, senior consultant status, and ratio of occupation on ICU. This table presents the results of the respective single-item statistical analysis of the MR2′s items on self-confidence and their association with certification in Intensive care, senior consultant status, and ratio of occupation on ICU. [file 13104_2020_5010_MOESM1_ESM.docx]

| **Appendix A – Associations of the MR2’s items on self-confidence with certification in Intensive care, senior consultant status, and ratio of occupation on ICU** | | | | | | | | | | | | | | | | | | | |
| --- | --- | --- | --- | --- | --- | --- | --- | --- | --- | --- | --- | --- | --- | --- | --- | --- | --- | --- | --- |
| Self-confidence about… | **Certificate in Intensive Care (CIC)** | | | | | **Senior Consultant Status** | | | | | **Time spent on ICU** | | | | | | | | |
|  | **Non CIC**  **(Mean ± SD)** | **CIC**  **(Mean ± SD)** | **^a)^p** | **OR CIC (95% CI)** | **^b)^p-MLRM** | **Non Senior (Mean±SD)** | **Senior (Mean±SD)** | **^a)^p** | **OR Senior (95% CI)** | **^b)^p-MLRM** | **Non ICU Mean±SD)** | **ICU Mean±SD)** | **^a)^P** | **1-50% ICU OR (95% CI)** | **^b)^p-MLRM** | **50-100% OR (95% CI)** | **^b)^p-MLRM** | **100% OR (95% CI)** | **^b)^p-MLRM** |
| … the correct choice of microbiological diagnostics | 2.75±0.614 | 3.17±0.577 | <0.001** | 3.379 (1.794-8.023) | <0.001** | 2.84±0.577 | 3.05±0.680 | 0.002* | 0.690 (0.349–1.365) | 0.0286 | 2.63±0.632 | 3.12±0.557 | <0.001** | 2.402 (1.274-4.529) | 0.007* | 8.941 (2.573-31.073) | 0.001* | 8.78x10^8 | 0.997 |
| … the correct interpretation of microbiological results | 2.77±0.547 | 3.11±0.491 | <0.001** | 4.179 (1.869-9.346) | <0.001** | 2.81±0.551 | 3.05±0.551 | <0.001** | 1.116 (0.550-2.265) | 0.760 | 2.69±0.565 | 3.05±0.495 | <0.001 | 3.374 (1.644-6.923) | 0.001* | 2.261 (1.044-6.578) | 0.040* | 9.298 (2.089-41.388) | 0.003* |
| … the correct choice of the right antibiotic substance | 2.47±0.573 | 2.90±0.542 | <0.001** | 3.436 (1.885-6.262) | <0.001** | 2.52±0.565 | 2.83±0.605 | <0.001** | 1.515 (0.839-2.737) | 0.168 | 2.42±0.617 | 2.80±0.545 | <0.001** | 3.541 (1.941-6.461) | <0.001** | 3.352 (1.532-7.334) | 0.002* | 3.571 (1.508-8.452) | 0.004* |
| … the correct decision whether oral or intravenous antibiotic therapy is indicated | 2.75±0.665 | 3.06±0.655 | <0.001** | 1.553 (0.858-2.812) | 0.146 | 2.77±0.641 | 3.03±0.699 | <0.001** | 1.399 (0.779-2.512) | 0.261 | 2.63±0.608 | 3.04±0.670 | <0.001 | 1.852 (1.043-3.291) | 0.036* | 2.640 (1.188-5.870) | 0.017* | 4.380 (1.594-12.035) | 0.004* |
| … the correct decision regarding dose, frequency and duration of antibiotics | 2.56±0.651 | 2.89±0.607 | <0.001** | 1.919 (1.100-3.349) | 0.022* | 2.60±0.615 | 2.84±0.678 | 0.001* | 1.159 (0.668-2.012) | 0.599 | 2.49±0.667 | 2.83±0.611 | <0.001** | 1.712 (0.988-2.966) | 0.055 | 2.666 (1.259-5.646) | 0.010* | 3.150 (1.361 -7.286) | 0.007* |
| … the indication of antibiotic combination therapy | 2.25±0.623 | 2.73±0.594 | <0.001** | 2.271 (1.330-3.877) | 0.003* | 2.30±0.605 | 2.68±0.655 | <0.001** | 1.678 (0.980-2.872) | 0.059 | 2.23±0.608 | 2.60±0.641 | <0.001** | 2.149 (1.220-3.783) | 0.008* | 2.565 (1.263-5.210) | 0.009* | 3.590 (1.658-7.769) | 0.001* |
| Results from the comparison of means and the logistic regression adjusted for the following criteria 1) additional certification in intensive care, 2) senior consultant status, 3) work on the intensive care unit within 12 preceding months, 4) self-contained anti-infective medication during 7 preceding workdays, 5) participants gender; Item-wise comparisons have been computed using the Kruskal-Wallis Test for ordinal variables; it compares the categories (very unconfident<unconfident<confident<very confident). The LRM compare self-confidence levels *very unconfident* and *unconfident* vs *confident* and *very confident*. The German physicians can obtain an additional certification post residency after one additional year of full-time work on an intensive care unit. a) p-values for unadjusted comparisons; b) p-value from the logistic regression model Abbreviations: SD, standard deviation; OR, Odd’s ratio; CI, confidence interval; P-LRM, p-values gathered from the LRM; *, p<0.05; **, p<0.001. Transcript following the translation of the German MR2-survey in Lebentrau, Gilfrich (20). | | | | | | | | | | | | | | | | | | | |
