# Supplementary material for: The association of the anesthesiologist’s academic and educational status with self-confidence, self-rated knowledge and objective knowledge in rational antibiotic application
Source: BMC Res Notes. 2020 Mar 18;13:161. doi: 10.1186/s13104-020-05010-8 (PMC7079461; doi:10.1186/s13104-020-05010-8)
Supplement: Supplementary file 2 — Additional file 2: Table S2. Associations of the MR2’s items on self-rated knowledge with certification in Intensive care, senior consultant status, and ratio of occupation on ICU. This table presents the results of the respective single-item statistical analysis of the MR2’s items on self-rated knowledge and their association with certification in Intensive care, senior consultant status, and ratio of occupation on ICU. [file 13104_2020_5010_MOESM2_ESM.docx]

| **Appendix B – Associations of the MR2’s items on self-rated knowledge with certification in Intensive care, senior consultant status, and ratio of occupation on ICU** | | | | | | | | | | | | | | | | | | | | |
| --- | --- | --- | --- | --- | --- | --- | --- | --- | --- | --- | --- | --- | --- | --- | --- | --- | --- | --- | --- | --- |
| Self-rated knowledge about… | **Certificate in Intensive Care (CIC)** | | | | | **Senior Consultant Status** | | | | | **Time spent on ICU** | | | | | | | | |  |
|  | **Non CIC**  **(Mean ± SD)** | **CIC**  **(Mean ± SD)** | **^a)^p** | **OR CIC (95% CI)** | **^b)^p-MLRM** | **Non Senior (Mean±SD)** | **Senior (Mean±SD)** | **^a)^p** | **OR Senior (95% CI)** | **^b)^p-MLRM** | **Non ICU Mean±SD)** | **ICU Mean±SD)** | **^a)^P** | **1-50% ICU OR (95% CI)** | **^b)^p-MLRM** | **50-100% OR (95% CI)** | **^b)^p-MLRM** | **100% OR (95% CI)** | **^b)^p-MLRM** |  |
| … measures of Antibiotic Stewardship (ABS) | 2.07±0.853 | 2.68±0.807 | <0.001** | 2.585 (1.465-4.560) | 0.001* | 2.18±0.812 | 2.53±0.948 | <0.001** | 1.516 (0.861-2.669) | 0.149 | 1.94±0.826 | 2.56±0.840 | <0.001** | 2.343 (1.295-4.238) | 0.005* | 3.749 (1.794-7.835) | <0.001** | 3.653 (1.629-8.196) | 0.002* |  |
| … local resistance patterns | 2.12±0.673 | 2.61±0737 | <0.001** | 2.918 (1.677-5.079) | <0.001** | 2.14±0.671 | 2.58±0.762 | <0.001** | 1.947 (1.125-3.369) | 0.017* | 2.05±0.695 | 2.50±0.717 | <0.001** | 2.619 (1.446-4.743) | 0.001* | 1.673 (0.803-3.486) | 0.169 | 5.546 (2.421-12.704) | <0.001** |  |
| … amount of local antibiotic prescribing | 1.90±0.755 | 2.18±0.864 | 0.002* | 0.932 (0.504-1.726) | 0.824 | 1.84±0.696 | 2.26±0.898 | <0.001** | 3.776 (2.041-6.988) | <0.001** | 1.74±0.714 | 2.19±0.828 | <0.001** | 3.539 (1.758-7.128) | <0.001** | 3.805 (1.660-8.721) | 0.002* | 4.190 (1.805-9.727) | 0.001* |  |
| … indications of MRSA-screening | 3.11±0.716 | 3.44±0.671 | <0.001** | 2.490 (1.096-5.659) | 0.029* | 3.19±0.685 | 3.33±0.750 | 0.025* | 0.539 (0.255-1.138) | 0.105 | 3.02±0.738 | 3.39±0.665 | <0.001** | 2.043 (0.959-4.353) | 0.064 | 5.165 (1.158-23.032) | 0.031* | 2.990 (0.834-10.714) | 0.093 |  |
| … indications of MDRGN-screening | 2.97±0.786 | 3.37±0.695 | <0.001** | 2.687 (1.270-5.685) | 0.010* | 3.04±0.764 | 3.26±0.772 | 0.004* | 0.932 (0.469-1.850) | 0.840 | 2.81±0.824 | 3.33±0.670 | <0.001** | 2.767 (1.389-5.512) | 0.004* | 3.582 (1.287-9.970) | 0.015* | 7.075 (1.603-31.236) | 0.010* |  |
| … patient cohort with need for isolation | 3.13±0.608 | 3.44±0.47 | <0.001** | 1.711 (0.600-4.877) | 0.315 | 3.19±0.581 | 3.36±0.646 | 0.002* | 0.778 (0.293-2.067) | 0.615 | 3.07±0.625 | 3.38±0.580 | <0.001** | 2.781 (0.964-8.025) | 0.059 | 3.066 (0.658-14.284) | 0.154 | 5.195 (0.651-41.488) | 0.120 |  |
| … the definitions of 3-MDRGN and 4-MDRGN | 3.33±0.685 | 3.68±0.494 | <0.001** | 6.648 (1.365-32.374) | 0.019* | 3.44±0.636 | 3.54±0.629 | 0.100 | 0.740 (0.257-2.125) | 0.575 | 3.31±0.696 | 3.59±0.570 | <0.001** | 2.274 (0.768-6.736) | 0.138 | 1.25x10^8 | 0.997 | 4.172 (0.516-33.736) | 0.180 |  |
| … hygiene activities and hygiene standards in the hospital | 3.20±0.594 | 3.34±0.528 | 0.044* | 2.845 (0.774-10.460) | 0.116 | 3.19±0.560 | 3.36±0.570 | 0.004* | 0.714 (0.234-2.179) | 0.554 | 3.20±0.596 | 3.30±0.551 | 0.127 | 1.121 (0.366-3.431) | 0.842 | 1.549 (0.305-7.874) | 0.598 | 2.873 (0.337-24.465) | 0.334 |  |
| … the current rules for hand disinfection | 3.62±0.552 | 3.74±0.469 | 0.037± | 2.272 (0.338-15.288) | 0.399 | 3.64±0.520 | 3.71±0.522 | 0.129 | 0.749 (0.130-4.318( | 0.746 | 3.65±0.510 | 3.69±0.528 | 0.312 | 0.388 (0.065-2.310) | 0.298 | 1.48x10^7 | 0.997 | 0.379 (0.030-4.778) | 0.453 |  |
| … the possibilities for success monitoring of sufficient hygiene measures and hygiene standards | 2.42±0.860 | 2.66±0.849 | 0.007* | 1.132 (0.676-1.896) | 0.637 | 2.36±0.851 | 2.73±0.835 | <0.001** | 1.964 (1.176-3.278( | 0.010* | 2.28±0.843 | 2.67±0.842 | <0.001** | 1.642 (0.967-2.788) | 0.066 | 2.367 (1.211-4.627) | 0.012* | 3.414 (1.603-7.270) | 0.001* |  |
| … the DART 2020 initiative (*German-Antimicrobial-Resistance-Strategy* by the Federal ministry of health) | 1.60±0.725 | 1.86±0.750 | <0.001** | 1.000 (0.489-2.044) | 0.999 | 1.59±0.677 | 1.89±0.799 | <0.001** | 2.268 (1.110-4.633) | 0.025* | 1.49±0.632 | 1.86±0.779 | <0.001** | 3.959 (1.656-9.512) | 0.002* | 4.502 (1.674-12.108) | 0.003* | 3.151 (1.113-8.922) | 0.031* |  |
| … the c*hoosing-wisely-initiative* (by the German society of infectious diseases, DGI) | 1.51±0.706 | 1.90±0.838 | <0.001** | 1.676 (0.830-3.383) | 0.150 | 1.53±0.711 | 1.87±0.851 | <0.001** | 1.789 (0.891-3.593) | 0.102 | 1.43±0.617 | 1.83±0.844 | <0.001** | 3.292 (1.419-7.637) | 0.006* | 3.720 (1.438-9.622) | 0.007* | 2.846 (1.044-7.754) | 0.041 |  |
| … obligations to report and responsibilities for reporting certain multidrug-resistant pathogens | 2.42±0.755 | 2.77±0.708 | <0.001** | 1.648 (0.974-2.787) | 0.063 | 2.44±0.688 | 2.75±0.807 | <0.001** | 1.611 (0.955-2.718) | 0.074 | 2.29±0.761 | 2.74±0.699 | <0.001** | 2.045 (1.197-3.492) | 0.009* | 2.489 (1.256-4.930) | 0.009* | 4.163 (1.860-9.317) | 0.001* |  |
| … mixing and cycling antibiotic treatment regimes | 1.96±0.725 | 2.50±0.776 | <0.001** | 2.340 (1.333-4.108) | 0.003* | 2.00±0.690 | 2.46±0.851 | <0.001** | 2.463 (1.397-4.341) | 0.002* | 1.85±0.676 | 2.40±0.788 | <0.001** | 3.111 (1.641-5.897) | 0.001* | 4.050 (1.892-8.669) | <0.001 | 6.654 (2.946-15.028) | <0.001** |  |
| … the procedure when infections with *Clostridium difficile* are identified | 2.94±0.702 | 3.30±0.668 | <0.001** | 3.572 (1.583-8.059) | 0.002* | 3.03±0.629 | 3.18±0.804 | 0.015 | 0.405 (0.195-0.840) | 0.015* | 2.74±0.720 | 3.31±0.614 | <0.001** | 3.779 (1.825-7.827) | <0.001** | 9.997 (2.283-43.773) | 0.002* | 6.65x10^7 | 0.997 |  |
| … the necessary actions to take when vancomycin resistant enterococcus is identified | 2.91±0.692 | 3.26±0.728 | <0.001** | 2.480 (1.186-5.189) | 0.016* | 2.96±0.663 | 3.19±0.793 | 0.001 | 0.878 (0.443-1.737) | 0.708 | 2.74±0.733 | 3.26±0.652 | <0.001** | 3.228 (1.654.6.300) | 0.001* | 8.001 (2.313-27.673) | 0.001* | 18.982 (2.502-143.992) | 0.004* |  |
| Results from the comparison of means and the logistic regression adjusted for the following criteria 1) additional certification in intensive care, 2) senior consultant status, 3) work on the intensive care unit within 12 preceding months, 4) self-contained anti-infective medication during 7 preceding workdays, 5) participants gender; Item-wise comparisons have been computed using the Kruskal-Wallis Test for ordinal variables (the compared categories were no knowledge<little knowledge<knowledge<full knowledge). The LRM compare self-rated knowledge levels *no knowledge* and *little knowledge* vs. *knowledge* and *full knowledge*. The German physicians can obtain an additional certification post residency after one additional year of full-time work on an intensive care unit. a) p-values for unadjusted comparisons; b) p-value from the logistic regression model Abbreviations: SD, standard deviation; OR, Odd’s ratio; CI, confidence interval; P-LRM, p-values gathered from the LRM; *, p<0.05; **, p<0.001. Transcript following the translation of the German MR2-survey in Lebentrau, Gilfrich (20). | | | | | | | | | | | | | | | | | | | |  |
