# Supplementary material for: The association of the anesthesiologist’s academic and educational status with self-confidence, self-rated knowledge and objective knowledge in rational antibiotic application
Source: BMC Res Notes. 2020 Mar 18;13:161. doi: 10.1186/s13104-020-05010-8 (PMC7079461; doi:10.1186/s13104-020-05010-8)
Supplement: Supplementary file 3 — Additional file 3: Table S3. Associations of the MR2’s items on objective knowledge with certification in Intensive care, senior consultant status, and ratio of occupation on ICU. This table presents the results of the respective single-item statistical analysis of the MR2’s items on objective knowledge and their association with certification in Intensive care, senior consultant status, and ratio of occupation on ICU. [file 13104_2020_5010_MOESM3_ESM.docx]

| **Appendix C – Associations of the MR2’s items on objective knowledge with certification in Intensive care, senior consultant status, and ratio of occupation on ICU** | | | | | | | | | | | | | | | | | | | | | |
| --- | --- | --- | --- | --- | --- | --- | --- | --- | --- | --- | --- | --- | --- | --- | --- | --- | --- | --- | --- | --- | --- |
|  | **Certificate in Intensive Care (CIC)** | | | | | **Senior Consultant Status** | | | | | | **Time spent on ICU** | | | | | | | | | |
|  | **Non CIC**  **(Mean ± SD)** | **CIC**  **(Mean ± SD)** | **^a)^p** | **OR CIC (95% CI)** | **^b)^p-MLRM** | | **Non Senior (Mean±SD)** | **Senior (Mean±SD)** | **^a)^p** | **OR Senior (95% CI)** | **^b)^p-MLRM** | | **Non ICU Mean±SD)** | **ICU Mean±SD)** | **^a)^P** | **1-50% ICU OR (95% CI)** | **^b)^p-MLRM** | **50-100% OR (95% CI)** | **^b)^p-MLRM** | **100% OR (95% CI)** | **^b)^p-MLRM** |
| **The ciprofloxacin resistance-rates of Escherichia coli in the participants hospital (calculated from the categories <5, 5-20, 21-40, 41-60, >60% and matched with the real resistance-rates)** | | | | | | | | | | | | | | | | | | | | | |
| correctly estimated  no answer  underestimated  overestimated | 41 (20%)  54 (26%)  88 (43%)  22 (11%) | 52 (34%)  16 (10%)  71 (46%)  16 (10%) | 0.424 | 1.708(0.969-3.040) | 0.069 | | 47 (23%)  46 (22%)  93 /45%)  20 (10%) | 46 (30%  24 (15%)  66 (43%)  18 (12%) | 0.691 | 0.916(0.515-1.629) | 0.764 | | 25 (18%)  39 (29%)  58 (43%)  14 (10%) | 68 (34%)  31 (15%)  102 (50%)  2 (1%)) | 0.589 | 1.484(0.797-3.520) | 0.213 | 1.675(0.797-3.520) | 0.173 | 2.401(1.125-5.126) | 0.024 |
| **The MRSA-rates in the participants hospital (calculated from the categories <5, 5-20, 21-40, 41-60, >60% and matched with the real resistance-rates)** | | | | | | | | | | | | | | | | | | | | | |
| correctly estimated  no answer  underestimated  overestimated | 124 (61%)  15 (7%)  39 (19%)  27 (13%) | 102 (65%)  6 (4%)  41 (26%)  7 (5%) | 0.220 | 1.141 (0.679-1.917) | 0.618 | | 125 (61%)  14 (7%)  42 (20%)  25 (12%) | 100 (65%)  7 (4%)  38 (25%)  9 (6%) | 0.304 | 1.088 (0.652-1.815) | 0.746 | | 83 (61%)  14 (10%)  24 (18%)  15 (11%) | 143 (63%)  7 (3%)  56 (25%)  19 (8%) | 0.819 | 0.996 (0.585-1.695) | 0.987 | 1.033 (0.530-2.012) | 0.925 | 1.257 (0.613-2.576) | 0.532 |
| **Guideline adherent choice of perioperative antibiotic prophylaxis for knee-arthroplasty in a healthy patient (no contraindications, correct choice is option 6)** | | | | | | | | | | | | | | | | | | | | | |
| Ciprofloxacin once (Option 1)  Ceftriaxone once (Option 2)  Penicillin G once (Option 3)  Vancomycin once (Option 4)  Cefazolin for 3 days (Option 5)  Cefazolin once (Option 6) | 5 (3%)  41 (20%)  1 (1%)  2 (1%)  9 (4%)  142 (71%) | 1 (1%)  26 (17%)  0 (0%)  1 (1%)  1 (1%)  122 (80%) | 0.048* | 1.612 (0.857-2.968) | 0.125 | | 5 (2%)  41 (20%)  1 (1%)  2 (1%)  6 (3%)  145 (73%) | 1 (1%)  26 (17%)  0 (0%)  1 (1%)  4 3%)  118 (78%) | 0.171 | 1.072 (0.595-1.932) | 0.818 | | 2 (1%)  29 (22%)  1 (1%)  0 (0%)  6 (4%)  95 (72%) | 4 (2%)  38 (17%)  0 (0%)  3 (1%)  4 (2%)  169 (78%) | 0.238 | 1.645 (0.873-3.096) | 0.123 | 1.108 (0.518-2.370) | 0.791 | 1.086 (0.493-2.389) | 0.838 |
| **Guideline adherent choice of perioperative antibiotic prophylaxis for colorectal surgery in a healthy patient**  **(no contraindications, correct choice is option 1)** | | | | | | | | | | | | | | | | | | | | | |
| Cefuroxime/Metronidazole once (Option 1)  Metronidazole once (Option 2)  Meropenem once (Option 3)  Piperacillin/Tazobactam once (Option 4)  Penicillin G once (Option 5) | 186 (91%)  0 (0%)  0 (0%)  18 (9%)  0/ | 131 (85%)  0 (0%)  1 (1%)  22 (14%)  0 (0%) | 0.075 | 0.727 (0.331-1.596) | 0.427 | | 187 (91%)  0 (0%)  0 (0%)  18 (9%)  0 (0%) | 129 (85%)  0 (0%)  1 (1%))  22 (14%)  0 (0%) | 0.066 | 0.429 (0.192-0.961) | 0.040* | | 121 (90%)  0 (0%)  0 (0%)  13 (10%)  0 (0%) | 196 (87%)  0 (0%)  1 (1%)  27 (12%)  0 (0%) | 0.429 | 0.772 (0.319-1.869) | 0.567 | 0.247 (0.094-0.648) | 0.004* | 1.994 (0.405-9-813) | 0.396 |
| **Guidelines adherent choice of the optimal time for intravenous perioperative prophylaxis with cefazolin in a healthy patient without contraindications (correct choice is option 2)** | | | | | | | | | | | | | | | | | | | | | |
| Two hours prior to skin incision (Option 1)  One hour prior to skin incision (Option 2)  30 minutes past skin incision (Option 3)  With skin incision (Option 4)  Time does not matter (Option 5) | 1 (1%)  157 (78%)  34 (17%)  9 (4%)  0 (0%) | 3 (2%)  120 (78%)  26 (17%)  5 (3%)  0 (0%) | 0.560 | 1.147 (0.629-2.091) | 0.655 | | 2 (1%)  161 (80%  32 (16%)  7 (3%)  0 (0%) | 2 (1%)  115 (76%)  28 (18%)  7 (5%)  0 (0%) | 0.433 | 0.737 (0.409-1.329) | 0.310 | | 1 (1%)  107 (81%)  16 (12%)  8 (6%)  0 (0%) | 3 (1%)  170 (76%)  44 (20%)  6 (3%)  0 (0%) | 0.533 | 0.684 (0.367-1.273) | 0.230 | 0.685 (0.319-1.469) | 0.330 | 1.186 (0.495-2.842) | 0.702 |
| Results from the comparison of means and the logistic regression adjusted for the following criteria 1) additional certification in intensive care, 2) senior consultant status, 3) work on the intensive care unit within 12 preceding months, 4) self-contained anti-infective medication during 7 preceding workdays, 5) participants gender; Item-wise comparisons have been computed using the Chi-Square Test for categorial variables; it compares the incorrect categories and the correct answer. The LRM compare *correct answers* against individuals with *no* *answer* or *incorrect answer*. The German physicians can obtain an additional certification post residency after one additional year of full-time work on an intensive care unit. The results for the simple comparisons are provided as number and percentage within the group. a) p-values for unadjusted comparisons; b) p-value from the logistic regression model Abbreviations: OR, Odd’s ratio; CI, confidence interval; P-LRM, p-values gathered from the LRM; *, p<0.05; **, p<0.001. Transcript following the translation of the German MR2-survey in Lebentrau, Gilfrich (21) | | | | | | | | | | | | | | | | | | | | | |
